# Breaking the vicious circle—the Asthma Referral Identifier (ReferID) tool

**DOI:** 10.1038/s41533-022-00296-6

**Published:** 2022-10-08

**Authors:** Maarten Beekman, Julie Hales, Mona Al-Ahmad, Ricardo del Olmo, Tze Lee Tan

**Affiliations:** 1 AstraZeneca, The Hague, The Netherlands; 2 AstraZeneca, Cambridgeshire, England, UK; 3 Microbiology Department, Faculty of Medicine, Kuwait University, Kuwait, Kuwait; 4 Hospital María Ferrer and IDIM CR, Ciudad Autónoma de Buenos Aires, Buenos Aires, Argentina; 5 Yong Loo Lin School of Medicine, National University of Singapore, Singapore, Singapore

**Keywords:** Asthma, Respiratory tract diseases

## Abstract

Asthma is associated with a significant burden of disease, especially for patients with severe or uncontrolled asthma. Many patients with severe asthma still receive treatment in primary care settings and despite the availability of effective options, inadequate asthma treatment remains a concern, particularly the use of systemic corticosteroids to treat exacerbations and severe asthma. Around the world, many patients are stuck in a vicious circle of misdiagnosis, undertreatment, and poor understanding of disease severity and management. In this manuscript, we describe the development of The Asthma Referral Identifier (ReferID) tool, a simple, 4-item questionnaire that healthcare providers can use to help identify patients with uncontrolled and/or potentially severe asthma. ReferID was developed specifically for use in primary care clinics in low- and middle-income countries and other clinics, where the optimisation of asthma assessments and treatment recommended for countries with well-established healthcare systems, are not possible. ReferID was developed through an informal collaborative process involving international asthma experts as well as general practitioners, nurses, and specialists throughout the Asia Pacific, Latin America and Middle East regions, in conjunction with current evidence and treatment guidelines. In collaboration with local and regional partners around the world, the developers have adapted ReferID and translated it into 21 languages, and implementation is ongoing in 30 countries. ReferID has the potential to help break the vicious circle, improving disease outcomes and health-related quality of life for patients with asthma.

## Introduction

Chronic respiratory diseases were the third leading cause of death in 2017 [7% (95% confidence interval 6.8–7.2] of all deaths) behind cardiovascular diseases and cancer^1^. Asthma is the most common non-communicable respiratory disease and a major public health problem according to the World Health Organization and the Global Initiative for Asthma (GINA), with an estimated 339 million people affected globally^2,3^. Despite the availability of safe and effective therapies, preventable asthma deaths are an ongoing problem as a result of inadequate, inappropriate, or potentially harmful disease management^4–6^. Uncontrolled asthma affects ~30% of patients, whereas severe asthma affects ~10% of patients; among those in the latter group, 50–60% have severe, uncontrolled asthma^7–10^.

Although many patients with severe or uncontrolled asthma continue to receive treatment in primary care settings, certain patients would benefit from referral to an asthma specialist to undergo a detailed review to include the discussion of an asthma management plan. This review allows for a variety of currently unidentified barriers to successful asthma management to be appropriately identified. These include patients with symptoms or activity limitations despite adherence to preventer medicines, patients who require multiple bursts or long-term OCS, patients who continue to experience exacerbations, and patients who are over-reliant on their SABA, among others^5,11^. The burden of disease for patients with severe or uncontrolled asthma is considerable and includes an increased risk of asthma exacerbations, greater asthma-related morbidity, higher healthcare resource utilisation and economic burden, and reduced health-related quality of life^12–14^. Systemic corticosteroids (SCS) are frequently used to manage asthma exacerbations and as a result, ~20–60% of patients with severe asthma receive long-term treatment with SCS globally, despite the established risks of increased morbidity and mortality associated with both acute and chronic SCS use^15–17^.

**Fig. 1:**
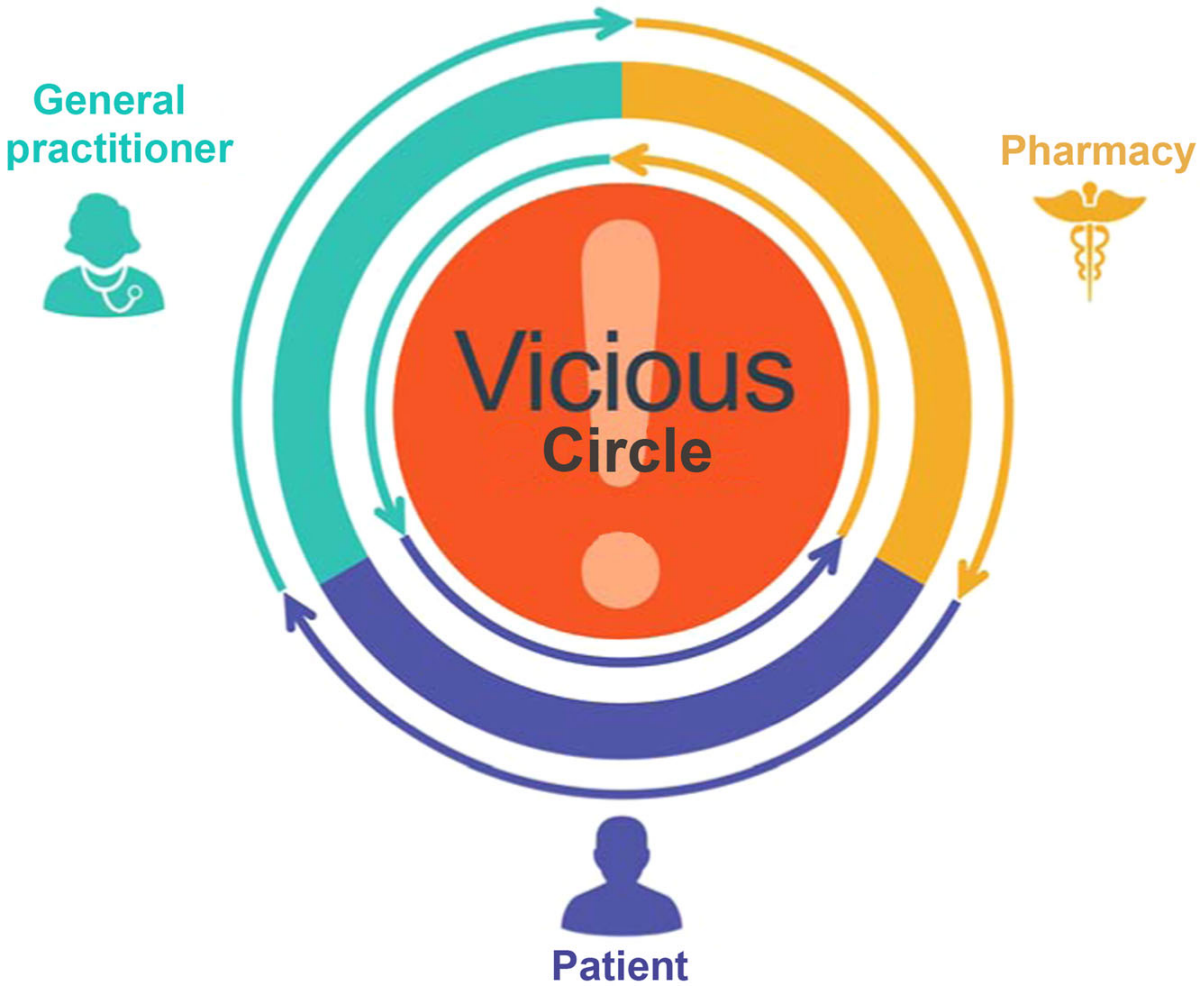
Vicious circle of care. Many patients with asthma are stuck in a vicious circle of misdiagnosis, inadequate treatment and poor understanding of disease severity and management.

In many regions, it can take years for a patient with asthma to receive an accurate asthma diagnosis or undergo a spirometry measurement, due to factors including social stigma and inaccessible/unaffordable care, especially in low- and middle-income countries. When these patients finally seek care for their symptoms, whether by purchasing over-the-counter medication at the pharmacy, seeing a general practitioner (GP), or visiting an emergency room or community hospital, they are often inappropriately diagnosed and inadequately treated with easily accessible medications rather than those ideally suited to treat their severity of disease^18–20^. Poor inhaler technique and poor treatment adherence can compound the problems arising from inadequate or inappropriate treatment, leading to poor disease outcomes and ultimately resulting in a vicious circle that can persist for years (Fig. 1)^2,6,21^. Evidence indicates that misperceptions about asthma control exist and thus, what many patients and physicians consider to be well-controlled asthma may in fact not be well-controlled at all, further contributing to the vicious circle^22–24^.

According to recent estimates across 18 countries, representing ~50% of the global population, patients spend 5 minutes or less with their primary care physicians during office visits^25^. Given the diversity of diseases that GPs diagnose and treat, in-depth knowledge of specific diseases, such as asthma, is often lacking and there is insufficient time to review key aspects of disease management; this challenge has been one of the main reasons for the development and growth of novel clinician support tools in low- and middle-income countries^26,27^. Furthermore, up-to-date local and national guidelines for respiratory diseases in general, and asthma in particular, may not be available or easily accessible in low- and middle-income countries^2,28,29^. There is a substantial unmet need for a tool that can help identify patients with uncontrolled and/or potentially severe asthma, to ensure that patients are diagnosed and managed effectively by the appropriate healthcare providers (HCPs) following best-practice guidelines. The short visit time available for primary care visits in many countries necessitates that any such tool must be quick to administer and easy to use^4,5,11^. The objective of this article is to describe the development and rollout of a simple and concise tool, the Asthma Referral Identifier (hereafter referred to as ReferID), which aims to help identify patients with uncontrolled and/or potentially severe asthma with the ultimate goal of improving disease management and long-term patient outcomes.

## ReferID development

ReferID originated from the concept of the Asthma Patient Navigator (hereafter referred to as ReferID^+^)—a tool designed to facilitate structured, comprehensive asthma consultations—conceived by David Jackson (Guy’s and St. Thomas’ NHS Trust and King’s College London, UK) for use in the UK National Health Service environment, subject to the successful piloting of the tool. The more comprehensive ReferID^+^ tool was designed to guide structured consultations and ensure that critical elements of the asthma review are covered, treatment is optimised, and patients with uncontrolled and/or potentially severe asthma receive appropriate, timely referrals to specialist clinics in secondary care. This ReferID^+^ tool will particularly be useful in countries with well-established healthcare systems. The ReferID tool described here was developed specifically for use in low- and middle-income countries and for cases where primary care clinics have substantial resource limitations and simply do not have the means to reassess inhaler technique, adherence, SABA overuse, or many of the other factors that frequently contribute to poor disease control and risks of future sub-optimal outcomes. In these settings, by referring patients for a specialist consultation, the specialist will be able to reassess the patient, optimise treatment, and, in many cases, refer the patient back to the primary care physician with improved asthma management and treatment options. We developed ReferID in collaboration with asthma experts from around the world. Both tools, ReferID^+^ and ReferID, have been developed in partnership with the PRECISION programme (supported by AstraZeneca), a global initiative to improve the care and outcomes of patients with severe and uncontrolled asthma by increasing access to healthcare and improving both the speed and quality of treatment^4^. During our initial collaborations on ReferID, key international asthma experts provided region-specific insights on obstacles to treating asthma in primary care settings in low- and middle-income countries and suggested opportunities for how ReferID could help overcome those obstacles (Table 1). During these discussions, common themes appeared across many regions: there is no clear pathway of care; patients are stuck in a vicious circle; and easily accessible guidelines for asthma management are not routinely available in primary care settings around the world.

**Table 1:** Key insights on issues and opportunities in the Asia Pacific, Latin America and Middle East Regions.

	Asia Pacific	Latin America	Middle East
Issues	• Lack of clear pathway in primary care • Vicious cycle, long wait for primary care, little follow-up • Frequent lack of medical records • Missing education about asthma and asthma treatment • Self-treatment with traditional medicine	• Vicious cycle, long wait for primary care, little follow-up • Short primary care consultations • Limited resources available for public primary care, especially for high-burden conditions	• Knowledge gaps about recognising and treating severe asthma • Lack of patient history data as clinics use different EMR systems and patients often switch clinics
Opportunities	• Break the vicious cycle; send patients to primary care sooner • Support patient retention in primary care • Support HCP decision-making • Improve asthma education for all stakeholders	• Upskill HCPs • Use consistent messaging • Improve asthma education for all stakeholders	• Upskill HCPs • Support HCP decision-making for referral • Improve asthma education for all stakeholders

*EMR* electronic medical record, *HCPs* healthcare providers.

To understand local patient and HCP needs, including the pathway of care, clinic workflows during new or follow-up patient visits, and potential pain points for end-use of ReferID in clinical practice, we spoke remotely with GPs and specialists throughout Asia Pacific, Latin America, and Middle East regions. We consulted with 17 HCPs, including 14 GPs and 3 specialists, from Singapore, Argentina and Kuwait. These HCPs were identified as colleagues of the collaborators who helped develop the pilot ReferID tool and were not selected through a systematic or objective process. Respondents had varying levels of experience and work settings, from public and private practices to urban and non-urban settings. These informal HCP consultations included a card-sorting exercise, a survey about a pilot version of the ReferID tool, and conversations about their asthma consultation experience, daily clinic workflows, and the facilities available within their practice. These informal consultations were designed to gain functional insights on the user experience with the pilot ReferID tool and HCP-perceived challenges in specific regions and practice settings. The consultations were not intended as a formal consensus-gathering exercise and were not designed to assess or validate specific verbiage for questions in the ReferID tool; as such, these consultations were waived for consent and ethics approval. Finally, there are currently no plans to perform a formal consultation process with additional HCPs from these countries.

**Fig. 2:**
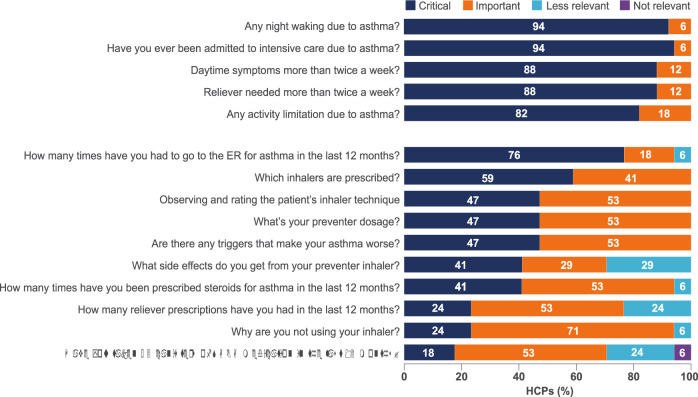
HCP prioritisation of the five most critical and ten other potential asthma assessment questions (*N* = 17 HCPs). ER emergency room, HCPs healthcare providers, SABA short-acting β_2_-agonists.

During the card-sorting exercise, which was used to guide HCP conversations through specific themes, HCPs were asked to rate 20 asthma assessment questions according to priority for identifying uncontrolled or potentially severe asthma. HCPs ranked questions about asthma symptoms and exacerbations as those most helpful for understanding whether a patient has uncontrolled or severe disease, and priority rankings for the five most critical questions were generally consistent across the group (Fig. 2). There was less agreement about the relevance of other potential assessment questions related to patient risk factors (asthma triggers), patient physical characteristics (e.g., age, sex, height and weight), treatment adherence, and inhaler technique (Fig. 2). Following the card-sorting exercises, informal surveys were used to assess HCPs’ opinions about a pilot ReferID, to optimise form and function for the final ReferID tool. Most HCPs (82%) wanted to complete some or all of the assessment questions themselves, and most (82%) preferred a digital format, although some HCPs (18%) wanted the final tool in a paper format as well (Table 2). A majority of HCPs (65%) preferred that patients respond to assessment questions in front of them rather than in the clinic waiting room before the appointment (35%). Feedback from the card-sorting exercises, informal surveys and conversations were synthesised to identify challenges to asthma treatment in the Asia Pacific, Latin America and Middle East regions.

**Table 2:** HCP survey responses about the ideal ReferID format.

Survey question	Percentage of HCPs (*N* = 17)
Preferred primary user	
Patient answers questions on their own	18%
GP asks the patient each question	41%
Specialist asks the patient each question	0%
Mixed: patient completes some; HCP helps with the rest	41%
Preferred primary context of use	
In front of the HCP	65%
In the clinic waiting room	35%
Preferred primary format	
HCPs’ work laptop	41%
Patient’s mobile device	29%
HCP’s mobile device	12%
Paper	18%

*GP* general practitioner, *HCP* healthcare provider.

Key challenges in asthma care involve asthma seasonality, patient attitudes, patient education and barriers to care. Asthma burden is seasonal in many countries, and GPs see an influx of patients during certain peak months or seasons, for example, with increased dust flareups in spring and summer in the Middle East, during the winter flu season in South America, and with reductions in air quality in Singapore due to uncontrolled forest fires in neighbouring countries^30–34^. Concomitant with the seasonal nature of asthma are patients’ attitudes about the disease, which are largely reactive, meaning that patients may wait to visit their HCPs until they have symptoms, or they might skip follow-up appointments if their asthma improves (furthering patients’ misperceptions about the use of asthma preventers versus relievers, for example). Although the pathway of care is unclear in many regions, most HCPs said that primary care plays an important role in diagnosing and treating patients with asthma. HCPs in all regions stressed the significance of upskilling GPs and educating patients about asthma, especially basic asthma awareness, critical aspects of effective asthma management (i.e., inhaler technique and treatment adherence), and the importance of long-term care. In many regions, patients fail to seek consistent treatment for reasons outside their control, such as financial barriers and long wait times for public clinics; GPs and specialists alike suggested these constraints should be considered in the content and design of the final tool.

HCPs also provided region-specific insights on opportunities to improve patient outcomes by optimising specific aspects of the pathway of care, from the initial asthma diagnosis through treatment, follow-up asthma consultations, and potential referrals to a specialist. HCPs felt that the key factors for obtaining an accurate asthma diagnosis were the patient’s medical history, a physical examination, data from peak flow metres, responses to verbal questions, and guideline criteria. HCPs in all regions acknowledged the substantial utility of spirometry tests when making an asthma diagnosis; however, the tools are not always available in GP clinics in resource-limited areas, and respondents were concerned that including spirometry measurements in the ReferID algorithm might prevent widespread adoption of the tool. With respect to optimising treatment, HCPs highlighted the need to educate patients about inhaler technique, treatment adherence, and recommended roles for preventive and reliever medicines (i.e., consistent use of preventers to avoid overuse of short-acting beta_2_-agonists). Many HCPs reported that follow-up visits are vital to improving asthma treatment, particularly when considering whether to refer the patient to a specialist and for continued care following the specialist visit.

Most of the GPs, specialists, and international experts who were consulted about the pilot ReferID, agreed that the tool should be quick to complete and use the minimum questions necessary to accurately and consistently identify patients with uncontrolled and/or potentially severe asthma. This was particularly true for regions where HCPs may have limited time for patient consultation and lack specialised asthma knowledge. Indeed, beyond the concise assessment questions, HCPs wanted the flexibility to use ReferID for a more in-depth consultation if time allows, with access to patient education resources, referral recommendations from the GINA report, and resources to guide treatment optimisation^2^. Feedback from informal conversations with HCPs consistently underscored the importance of a format that is conducive to a back-and-forth patient-provider conversation and a shared decision-making process. HCPs recommended phrasing questions in layman’s terms, with local cultural context to support patients’ comprehension, so that HCPs could focus on the patient and effortlessly switch between interacting with the ReferID tool and conversing with the patient. The findings from this informal, user-centric qualitative research provided valuable insight on the ideal form and function of the ReferID tool. These insights are being used to translate and scale ReferID for rollout in countries around the world.

## ReferID content

ReferID is a simple and concise tool that HCPs can use to help quickly identify patients who have uncontrolled and/or potentially severe asthma that may benefit from an asthma review by a specialist. ReferID consists of four questions, resulting in a yes/no recommendation for a specialist review. Following the recommendation, users have the option to access additional guidelines-based strategies for optimising treatment. The four questions and associated rationale in ReferID are listed in Table 3^2,3,12–14,16,17,35,36^. These questions were developed in collaboration with international asthma experts, based on informal feedback from GPs and asthma specialists around the world, in conjunction with GINA recommendations and current peer-reviewed research, the foundations of which are briefly summarised in Table 3^2^. ReferID is readily scalable for use in multiple world regions and languages. It is available in both digital and paper formats, and the paper format includes a QR code, which links to online versions (Supplementary Fig. 1).

**Table 3:** ReferID questions.

Question	Rationale
Has the patient used 2 or more courses of SCS and/or is using maintenance SCS therapy over the past 12 months?	• SCSs have been associated with both acute and long-term adverse effects^16,17^ • SCS overuse is a driver of increased morbidity and mortality^16,17^
Has the patient had 2 or more emergency attendances/unscheduled visits due to asthma over the past 12 months?	• Uncontrolled asthma is associated with a higher disease burden, including increased asthma-related morbidity and increased risk for asthma exacerbations, emergency department visits, hospital admissions, and death^12–14^
Has the patient ever been intubated or admitted to an ICU or a high-dependency unit due to their asthma?	• GINA recommends a specialist referral for: ○ Patients with uncontrolled symptoms or frequent exacerbations^2^ ○ Patients with any risk factors for asthma-related death, which includes anaphylaxis or confirmed food allergy in a patient with asthma, or a prior asthma attack requiring ICU admission or mechanical ventilation^2^
Has the patient used 3 or more canisters of SABAs in past 12 months?	• SABA overuse is associated with increased risk of asthma exacerbations, which can be life-threatening and debilitating for patients, and which may be an indicator of uncontrolled or poorly controlled asthma^2,35,36^

*GINA* Global Initiative for Asthma, *ICU* intensive care unit, *SABA* short-acting β_2_-agonists, *SCS*, systemic corticosteroid.

## ReferID launch

**Fig. 3:**
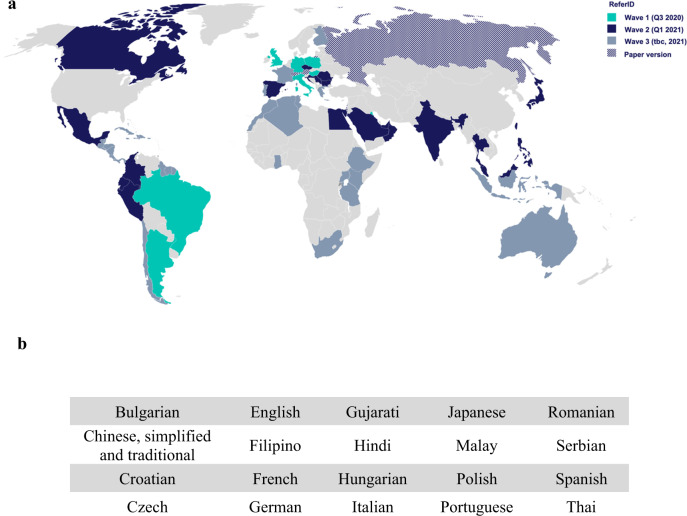
Countries participating in the ReferID launch and their associated languages. **a** Map showing countries involved in the launch. **b** Languages currently available in the ReferID tool.

ReferID has been adapted and translated into 21 languages, and the rollout is ongoing in 30 countries across Asia, Africa, Europe, North America, and South America (Fig. 3). In regions where ReferID has been released, the optimal outcome is that patients who are referred to specialists receive an accurate asthma diagnosis, a treatment plan, and education about their asthma. The vast majority of patients are referred back to their GP, whereas patients diagnosed with severe asthma (GINA Step 4/5) remain in the care of a specialist. Collaboration with local and regional partners, including professional societies, pharmacists, public health officials, governments, and community leaders, has been critical for the optimal rollout of ReferID in each country.

For example, ReferID was launched in Singapore in 2020 through partnerships with the College of Family Physicians Singapore, *Singapore Medical Journal*, and the Singapore Medical Association. This effort resulted in a peer-reviewed publication in *The Singapore Family Physician* as well as the Referral Expansion for Severe asthma “RESET” initiative, which recently began as an effort to reset the asthma circuit by improving appropriate referrals between GPs and specialists^37^. ReferID rollout efforts were launched in India at the end of 2020; current partnerships include the Railway Hospital System, and launch meetings with additional partners are planned for 2021. In Poland, there is no standardised referral process for patients with asthma and thus, asthma assessments are not comprehensive. ReferID was rolled out in Poland in October 2020 and since then has been presented to the Polish Family Medicine Society and the Polish Allergy Society. The ReferID tool has received positive feedback and endorsements among partners in Poland and is planned for implementation via the AZdlaPOZ website (AstraZeneca for GPs). It will also be included on the website publishing the National Consultant in the Field of Family Medicine’s video introduction and recommendation. In Japan, Kuwait, Saudi Arabia and the United Arab Emirates, ReferID was launched in a paper format that was adapted to support GPs and patients; GPs complete ReferID as a paper handout and the patient then takes it with them to their appointment with the specialist. The paper version of ReferID is frequently distributed in networking meetings between GPs and specialists to broaden adoption of the ReferID tool, and, in some cases, specialists have begun including their name, institution, and test results.

In addition to the ReferID launches in the countries mentioned above, the more comprehensive ReferID^+^ tool is undergoing evaluation in London, United Kingdom, through the Optimisation of ASthma In those with uncontrolled Symptoms (OASIS) study (NCT04941001). The primary objective of OASIS is to evaluate the effectiveness of ReferID^+^ after 12 months in primary care by comparing asthma exacerbation rates among patients who receive asthma care versus patients who receive an asthma review with ReferID^+^. ReferID^+^ has also been adapted into the Asthma Optimiser tool, which was developed expressly for use in The Netherlands and is being evaluated in the prospective, observational, CAPTURE pilot study (NCT04456270). The CAPTURE study will evaluate the feasibility and utility of Asthma Optimiser in primary care in The Netherlands, where HCPs will use Asthma Optimiser in daily clinical practice. The primary outcome of CAPTURE will be the percentages of patients with inadequate asthma control, measured by the 6-item Asthma Control Questionnaire (ACQ-6)^38^.

## Discussion

ReferID is a simple and concise tool that HCPs can use to help quickly identify patients with uncontrolled and/or potentially severe asthma who may benefit from a review by a specialist. ReferID further upskills HCPs by providing access to patient-education resources and in-depth strategies for optimising treatment. ReferID was built on a digital platform to simplify the process of scaling and replicating implementation across the globe, especially in low- and middle-income regions. Collaborations with local partners have been critical for successful implementation, and these partnerships have significant potential to drive long-term improvements in asthma management. Nevertheless, there are a few obstacles to overcome. Asking GPs to refer their patients to a specialist can be contentious in some regions due to a lack of perceived potential for improvements in treatment, financial reasons, or misperceptions about additional treatment strategies.

In countries where digital technologies like electronic medical records, medical guidelines apps, and GP decision-support tools (e.g., GP Notebook) are widely used in primary care, such as the United Kingdom, Canada, and the Netherlands, there is a significant opportunity for ReferID and/or ReferID^+^ to synergise with existing digital platforms to further upskill HCPs while streamlining primary care visits^39^. In regions where digital capabilities are limited, or in clinics where GPs prefer a non-digital approach, ReferID is available in a physical paper version. Given the substantial economic burden of asthma, ReferID has significant potential to reduce healthcare resource utilisation and lower national and regional healthcare burdens of disease beyond its implementation in primary care clinics^40^. Furthermore, launching ReferID in other healthcare settings (e.g., pharmacies) in the future may yield additional benefits beyond those observed in the GP setting.

In conclusion, ReferID is a simple tool for HCPs to help detect uncontrolled and/or potentially severe asthma and break the vicious circle of inadequate treatment, poor outcomes, and poor treatment adherence. By doing so, ReferID will lead to better patient outcomes and improvements in health-related quality of life in regions throughout the world.

## Supplementary information

Supplemental Figure 1
