# Supplementary material for: Breaking the vicious circle—the Asthma Referral Identifier (ReferID) tool
Source: NPJ Prim Care Respir Med. 2022 Oct 8;32:40. doi: 10.1038/s41533-022-00296-6 (PMC9547879; doi:10.1038/s41533-022-00296-6)

SUPPLEMENTARY MATERIAL

Supplementary Figure 1. ReferID (A) and ReferID Additional Guidance (B) in Paper Format.

A)

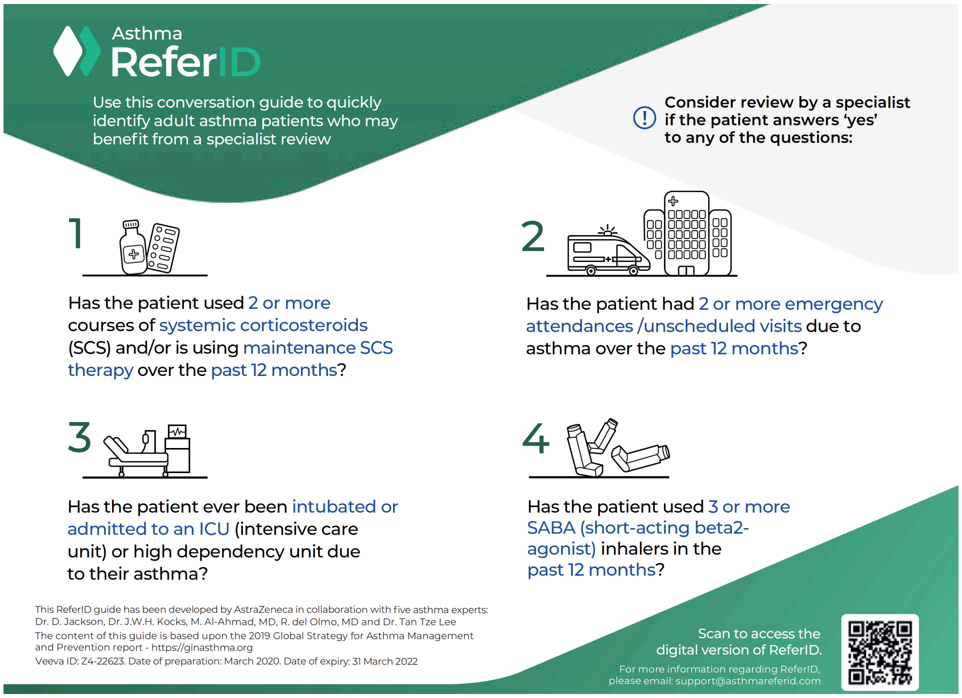

B)

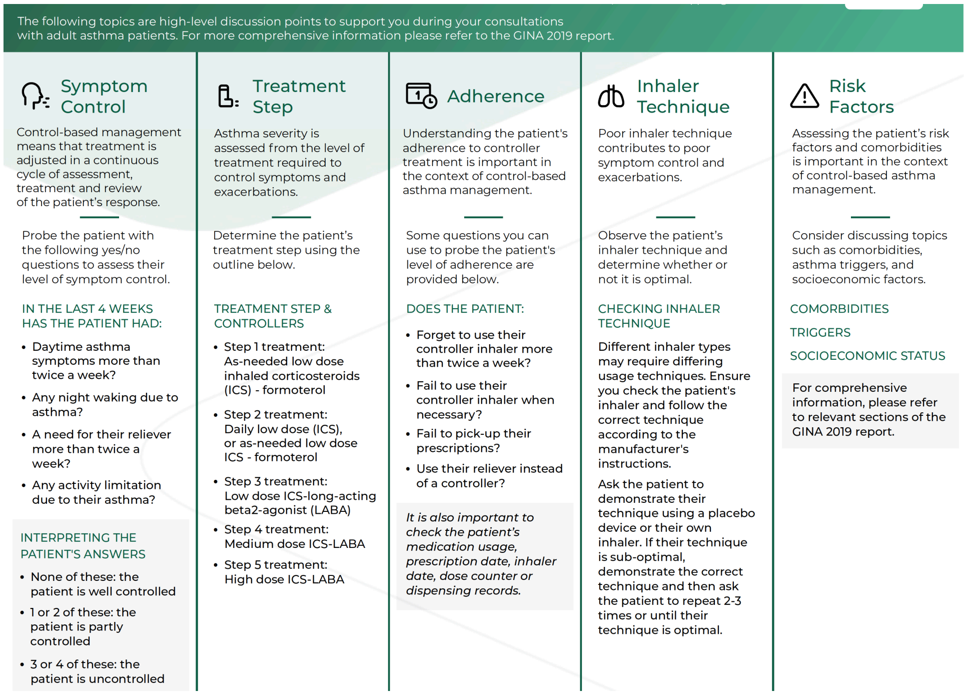

Supplement: Supplementary file 1 — Supplemental Figure 1 [file 41533_2022_296_MOESM1_ESM.pdf]
